# Heart Failure: Diagnosis, Management and Utilization

**DOI:** 10.3390/jcm5070062

**Published:** 2016-06-29

**Authors:** Arati A. Inamdar, Ajinkya C. Inamdar

**Affiliations:** 1 John Theurer Cancer Center, Hackensack University Medical Center, Hackensack, NJ 07601, USA; 2 Ansicht Scidel Inc., Edison, NJ 08837, USA; ajinkya.inamdar@gmail.com

**Keywords:** biomarker, heart failure, ICD 10, readmission, utilization

## Abstract

Despite the advancement in medicine, management of heart failure (HF), which usually presents as a disease syndrome, has been a challenge to healthcare providers. This is reflected by the relatively higher rate of readmissions along with increased mortality and morbidity associated with HF. In this review article, we first provide a general overview of types of HF pathogenesis and diagnostic features of HF including the crucial role of exercise in determining the severity of heart failure, the efficacy of therapeutic strategies and the morbidity/mortality of HF. We then discuss the quality control measures to prevent the growing readmission rates for HF. We also attempt to elucidate published and ongoing clinical trials for HF in an effort to evaluate the standard and novel therapeutic approaches, including stem cell and gene therapies, to reduce the morbidity and mortality. Finally, we discuss the appropriate utilization/documentation and medical coding based on the severity of the HF alone and with minor and major co-morbidities. We consider that this review provides an extensive overview of the HF in terms of disease pathophysiology, management and documentation for the general readers, as well as for the clinicians/physicians/hospitalists.

## 1. Introduction

### 1.1. Background

Heart failure (HF) is a clinical syndrome caused by structural and functional defects in myocardium resulting in impairment of ventricular filling or the ejection of blood. The most common cause for HF is reduced left ventricular myocardial function; however, dysfunction of the pericardium, myocardium, endocardium, heart valves or great vessels alone or in combination is also associated with HF. Some of the major pathogenic mechanisms leading to HF are increased hemodynamic overload, ischemia-related dysfunction, ventricular remodeling, excessive neuro-humoral stimulation, abnormal myocyte calcium cycling, excessive or inadequate proliferation of the extracellular matrix, accelerated apoptosis and genetic mutations [1].

### 1.2. Classification of HFs

Heart failure can be classified as predominantly left ventricular, right ventricular or biventricular based on the location of the deficit. Depending on the time of onset, HF is classified as acute or chronic. Clinically, it is typically classified into two major types based on the functional status of heart: heart failure with preserved ejection fraction (HFpEF) and heart failure with reduced ejection fraction (HFrEF). In patients with HFpEF who are mostly females and older adults, EF is usually more than 50%; the volume of the left-ventricular (LV) cavity is typically normal, but the LV wall is thickened and stiff; hence, the ratio of LV mass/end-diastolic volume is high [2]. HFpEF is further categorized as borderline HF if the EF stays between 41% and 49% and improved HF if EF is more than 40% [1]. In contrast, in patients with HFrEF, the LV cavity is typically dilated, and the ratio of LV mass/end-diastolic volume is either normal or reduced. At the cellular level, both cardiomyocyte diameter and the volume of myofibrils are higher in HFpEF than in HFrEF [1]. As far as treatment and outcome are concerned, patients with HFrEF respond favorably to the standard pharmacological treatment regimen and demonstrate better prognosis. In contrast, patients with HFpEF have not been shown to respond to standard pharmacological treatments, except for nitrates, and therefore, have a poor prognosis, especially during the decompensated phase of HF [2,3,4]. In addition, based on cardiac output, HF is also classified as high-output failure and low-output failure. High-output failure is an uncommon disorder characterized by an elevated resting cardiac index of greater than 2.5–4.0 L/min/m^2^ and low systemic vascular resistance. The common causes of high output failure are severe anemia, vascular shunting, hyperthyroidism and vitamin B1 deficiency. This occurs as a result of ineffective blood volume and pressure, which stimulate the sympathetic nervous system and renin-angiotensin-aldosterone system (RAAS), causing the release of antidiuretic hormone (ADH), which all together ultimately lead to ventricular enlargement, negative ventricular remodeling and HF. Low output failure is much more common than high-output failure and is characterized by insufficient forward cardiac output, particularly during times of increased metabolic demand. Left ventricular dysfunction due to large MI, right ventricular dysfunction due to an acute pulmonary embolus and biventricular dysfunction are important causes of low output failure. More recently, exercise intolerance in HFpEF is proposed to be due to a decrease in oxygen delivery to or impaired oxygen utilization by the exercising skeletal muscles. Oxygen utilization is being calculated as the arterial–venous oxygen content difference (A-VO_2_ Diff), rather than reduced cardiac output (CO) [5,6]. Considering the slowed down oxygen uptake kinetics in HF along with peripheral muscle function impairment, exercise rehabilitation seems to be a logical and essential factor in improving the inflammatory imbalance, relieving elevated cardiac filling pressures, restoring exercise capacity, quality of life and reducing morbidity and mortality associated with HF. Hence, exercise training, mostly high intensity as opposed to moderate, in HFpEF patients has been significantly shown to improve rate of oxygen consumption or VO_2_ without affecting endothelial function [7,8].

The New York Heart Association (NYHA) functional classification defines four functional classes as:

Class I: HF does not cause limitations to physical activity; ordinary physical activity does not cause symptoms.
Class II: HF causes slight limitations to physical activity; the patients are comfortable at rest, but ordinary physical activity results in HF symptoms.
Class III: HF causes marked limitations of physical activity; the patients are comfortable at rest, but less than ordinary activity causes symptoms of HF.
Class IV: HF patients are unable to carry on any physical activity without HF symptoms or have symptoms when at rest.

The American College of Cardiology/American Heart Association (ACC/AHA) staging system is defined by the following four stages:

Stage A: High risk of heart failure, but no structural heart disease or symptoms of heart failure;
Stage B: Structural heart disease, but no symptoms of heart failure;
Stage C: Structural heart disease and symptoms of heart failure;
Stage D: Refractory heart failure requiring specialized interventions.

## 2. Clinical Presentation of HF

The clinical presentation of HF comprises symptoms of shortness of breath (SOB)/dyspnea (sensitivity of 84%–100%, but a specificity of 17%–34%); orthopnea/SOB on lying own (sensitivity of 22%–50% and a specificity of 74%–77%); paroxysmal nocturnal dyspnea (sensitivity 39%–41%, specificity from 80%–84%); fatigue/weakness/lethargy (due to HF-induced circulation-related abnormalities in skeletal muscles); edema, abdominal distention and right hypochondrial pain (most likely due to right-sided heart failure with sensitivity and specificity of 23% and 80%, respectively) [9,10]. Due to compensatory mechanisms, early stages of HF lack specific signs; however, late stages of HF demonstrate the following signs: tachycardia (99% specificity and 7% sensitivity); pedal edema (93% specificity and 10% sensitivity); increased jugular venous pressure (JVP) (usually > 6 cm; specificity of 92% and sensitivity of 39%), abnormal lung sounds (crackles) (specificity of 78% and sensitivity of 60%); S3 gallop (specificity of 99% and sensitivity of 13%). Other signs, such as hepatojugular reflux and ascites, are not found frequently in HF, but have a specificity of 96% and 97%, while a sensitivity of 24% and 1%, respectively [11,12]. Recent research has uncovered the microvascular dysfunction and subsequent decrease in O_2_ supply or mismatch with the O_2_ supply vs. demand in HF patients. Therapeutic strategies to improve muscle microvascular and oxidative function via exercise training, anti-inflammatory and antioxidant agents have been proposed to be essential to provide better exercise tolerance and quality of life [13].

HF has primarily been recognized as a disease of the elderly population (>60 years) and is reported to affect about 2%–3% of people in the United States. Of these include 10% of males and 8% of females. Unfortunately, these numbers are on a gradual increase due to the on-going prevalence of HF with increasing age. In the USA itself, about more than three million physician visits per year have been accounted for patients with HF as the primary health issue. In 2013, the total number of HF patients were 5.1 million, and direct costs were equal to $32 billion; and this cost is being projected to increase by about three-fold by 2030 [14]. As of 2011, the estimated lifetime cost of HF per individual patient was $110,000/year, with more than three-fourths of this cost consumed by ‘in-hospital care’ [15]. Interestingly, the five-year mortality rate for HF was reviewed to be approximately 50%, which is significantly higher than that of some cancers [16]. Among Medicare patients, 30-day all-cause, risk-standardized mortality rates for HF are 10%–12%, while 30-day, all-cause, risk-standardized readmission rates after hospital discharge are 20%–25% [17]. There is indeed a slight decrease in HF-related mortality from 2000 to 2014. The age-adjusted rate for HF-related mortality was 105.4 per 100,000 population in 2000 and reached 84.0 per 100,000 in 2014. Similarly, the percentage of in-hospital HF-related deaths declined from 42.6% in 2000 to 30% in 2014 [18]. Furthermore, although in a nursing home or long-term care facility, the percentage of deaths have been decreased from 30.1% in 2000 to 26.7% in 2014, such deaths have increased in the patients in residence and in outpatient clinics or hospice care by about 10% and 7%, respectively. Although the prognosis of other cardiac conditions, such as acute coronary syndrome (ACS), severe hypertension, valvular and congenital heart diseases, has improved over the past decade, the prevalence of HF has increased in a relatively exponential manner [18]. An increase in the prevalence of co-morbid conditions and risk factors, such as increased body mass index (BMI), metabolic syndrome, elevated apolipoprotein B/apolipoprotein A ratio and cigarette smoking, in these populations with relatively increased life expectancy may be some of the reasons behind the increased prevalence of HF [19]. Furthermore, available treatment options for HF only offer symptomatic relief and lack definitive curative treatment for the affected heart. As far as hospitalization is concerned, acute decompensated heart failure (ADHF) is the most common form of heart failure that accounts for ~80% of hospitalizations related to heart failure [19]. The common causes of ADHF include non-adherence to medication or dietary restrictions; uncontrolled hypertension; acute coronary syndrome/ischemia; dysrhythmia/arrhythmias and COPD exacerbation; alcohol intoxication or excess; thyroid conditions; pregnancy; and other iatrogenic conditions, such as postoperative fluid replacement or administration of steroids or non-steroidal anti-inflammatory drugs; all directly or indirectly leading to the progression of the underlying disease [19].

The underlying pathogenesis of HF also involves silent inflammatory and immune-regulatory responses, the activation of which still has not been completely understood. It has been proposed that in HF, excessive neuroendocrine activation leads to the activation of neuro-hormones and pro-inflammatory cytokines following an initial cardiac insult. Many of these pro-inflammatory and anti-inflammatory cytokines and their receptors, released endotoxins, adhesion molecules, nitric oxide and reactive oxygen species have been associated with various pathological aspects of HF [20,21]. The pro-inflammatory cytokines include tumor necrosis factor-α (TNF-α), sTNFR19 (soluble tumor necrosis factor receptor 1/2), soluble Fas protein, TNF-α-related apoptosis-inducing ligand (TRAIL), interleukin 6, activin A, myeloperoxidase, pentraxin-3, regulated on activation, normal T cell expressed and secreted (RANTES), C reactive protein, monocyte chemotactic protein 1 (MCP1) and macrophage inflammatory protein 1-α (MIP-1-α) [22]. Many of these inflammatory markers (such as IL-6, TNF-α, CRP) have been found to be upregulated in HF patients, especially in the ADHF phase. In light of these findings, several clinical trials have been designed, and drugs targeting inflammatory markers, nitric oxides and reactive oxidative species, such as etanercept, infliximab, glucocorticoids, statins and anti-oxidants, are being tested [21]. A newer pathological mechanism “gut hypothesis of heart failure” has been proposed. Here, HF-associated decreased CO and alteration of systemic circulation which lead to reduced intestinal perfusion and mucosal ischemia, thus causing disruption in intestinal barrier, increased gut permeability, increased bacterial translocation and increased circulating endotoxins. This in turn contributes to the elevated pro-inflammatory response reported in patients with HF. For example, the fasting plasma trimethylamine-*N*-oxide (TMAO) is reported to be elevated in HF patients and has recently been correlated to higher long-term mortality risk independent of other HF risk factors [23]. For this reason, several strategies have been designed to retain the normal micro-biome and maintain metabolic homeostasis in HF patients [24].

## 3. Diagnosis of HF

The evaluation for HF is performed using various parameters: physical examination to determine the presence of clinical symptoms and signs, blood tests, including complete blood count, urinalysis, complete metabolic profile for levels of serum electrolytes (including calcium and magnesium), blood urea nitrogen, serum creatinine, glucose, fasting lipid profile, liver function tests and thyroid-stimulating hormone.

Other HF-specific laboratory tests (especially in patients with a high possibility of heart failure) include brain natriuretic peptide (BNP) with 70% sensitivity and 99% specificity and *N*-terminal proBNP (NT-proBNP) with 99% sensitivity and 85% specificity, the measurement which has been recommended both in outpatient and in the hospital settings [1]. BNP is a neuro-hormone, which is an activated form of proBNP, the 108-amino acid polypeptide precursor, stored as secretory granules in both ventricles and, to a lesser extent, in the atria. In response to volume expansion and pressure overload, proBNP is secreted into ventricles and breaks down into its two cleaved forms, the 76-peptide, biologically-inert *N*-terminal fragment, NT-proBNP, and the 32-peptide, biologically-active hormone BNP. NT-proBNP and BNP have clinical significance both as diagnostic and prognostic markers in the management of HF. During the diagnosis of HF, in patients presenting with acute dyspnea, BNP levels of less than 100 pg/mL have a 90% negative predictive value (NPV), and values of more than 500 pg/mL have an 81% positive predictive value (PPV) [25]. The BNP level is a strong predictor of risk of death and cardiovascular events in patients previously diagnosed with heart failure or cardiac dysfunction. It is to be remembered that elevated BNP levels have also been associated with renal failure, pulmonary embolism, pulmonary hypertension and chronic hypoxia while obese and overweight individuals have relatively lower BNP levels. Furthermore, there has been no clinically significant difference between BNP and NT-proBNP in terms of the diagnostic and prognostic values, except for the longer half-life time of NT-proBNP (72 h) as opposed to 4 h for BNP and that NT-pro-BNP levels are less affected by obesity [9,26]. A recent review by Simons et al. discussed the criteria and cut off values for the diagnosis, prognosis and treatment guidance [27]. Accordingly, single measurement of natriuretic peptides (BNP ≤ 100 pg/mL or NTproBNP ≤ 300 pg/mL) rules out HF clinically, while BNP ≥ 500 pg/mL or NTproBNP ≥ 1800 pg/mL has been proposed to have a relatively lower level of evidence in clinical settings. Nevertheless, both BNP and NT-proBNP levels aid in decisions regarding admission/ discharge and risk stratification for HF patients. Patients with BNP level of less than 200 pg/mL at admission have been associated with 2% mortality rate as opposed to 9% mortality rate seen in patients with admission BNP level of more than 200 pg/mL [28]. NT-proBNP level equal to or higher than 5000 pg/mL at admission has been shown to be associated with in-hospital mortality rate of 22.5% and longer length of stay in remaining surviving patients [29].

Biomarkers not only provide valuable information about the pathophysiology of the disease, but also shed light on the severity of ongoing disease. As far as biomarkers for HF are concerned, the National Academy of Clinical Biochemistry has set forth comparable goals in a consensus document stating that a biomarker in HF ideally enables clinicians to: (i) identify possible underlying (and potentially reversible) causes of HF; (ii) confirm the presence or absence of the HF syndrome; and (iii) estimate the severity of HF and the risk of disease progression.

Multiple biomarkers have been classified depending on their putative functional impact on cardiac myocytes and the resulting pathophysiological changes in patients with HF and include (a) myocyte stretch biomarkers; (b) myocyte necrosis biomarkers; (c) systemic inflammation biomarkers; (d) oxidative stress biomarkers; (e) extracellular matrix turnover biomarkers; (f) neuro-hormone biomarkers; and (g) biomarkers of extra-cardiac processes, such as renal function. The specific biomarkers are shown in Table 1 along with the underlying mechanisms leading to their expression in HF patients. The details of the commonly-used HF biomarkers and other emerging biomarkers are described in other review articles authored by Ahmad et al., 2012, Gaggin and Januzzi, 2012, and van Kimmenade et al., 2013 [30,31,32].

Other diagnostic tests for HF include chest X-ray, transthoracic echocardiography (TTE), computerized tomography (CT) scans and magnetic resonance imaging (MRI). Chest X-rays are useful in evaluating heart size, pulmonary congestion and to detect alternative cardio-pulmonary diseases that may cause or contribute to the patient’s symptoms. A ‘two-dimensional echocardiogram with Doppler’ is recommended for initial evaluation of patients presenting with HF. TTE is useful to assess ventricular function, size, wall thickness, wall motion and valve function. TTE also helps to determine the ejection fraction of the heart and thus helps in selecting the appropriate therapy. Furthermore, TTE helps to assess the mitral valve inflow pattern, the pulmonary venous inflow pattern, mitral annular velocity to precisely evaluate LV filling and the left atrial pressure of the dysfunctional heart. Other parameters include tricuspid valve regurgitant gradient coupled with the measurement of inferior vena caval diameter and its response during respiration, which provide estimates of systolic pulmonary artery pressure and central venous pressure [33]. Many of these abnormalities should be looked for because they carry importance from a prognostic stand point. Routine repeat assessment of ventricular function via TTE is desired when a patient presents with ADHF, but in the absence of altering clinical status or a change in treatment, intervention is not indicated [9,34]. Magnetic resonance imaging assesses LV volume and EF measurements comparable to that obtained with echocardiography. The additional information about myocardial perfusion, viability and fibrosis, which is obtained from MRI, can help identify HF etiology and assess prognosis. Magnetic resonance imaging also provides high anatomical resolution of all aspects of the heart and surrounding structure, leading to its recommended use in known or suspected congenital heart diseases [33]. Cardiac CT provides accurate assessment of cardiac structure and function, including the coronary arteries [35]. However, both cardiac CT and MRI lose accuracy in patients with high heart rates. Apart from these investigative approaches, the utility of cardiac catheterization and coronary angiography are necessary in patients with new onset heart failure and angina symptoms [9].

## 4. Predictors of Poor Outcome and High Mortality Rate

In HF patients, exercise intolerance characterized by the reduction in peak VO_2_/VO_2_ max capacity (VO_2_ max is the maximum intake of oxygen despite an increase in exercise intensity) has been considered as the primary predictor of mortality and morbidity [13,36]. In addition, higher age, increased blood urea nitrogen, creatinine and heart rate, lower systolic pressure and serum sodium, presence of dyspnea at rest, lack of long-term treatment with a β-blocker, male gender and lower body mass index and hemoglobin levels have been identified as independent predictors of mortality. The following values have been shown to predict the increased mortality in inpatient settings/hospitals [37,38,39,40].

- Serum urea >15 mmol/L
- Systolic blood pressure <115 mmHg
- Serum creatinine >2.72 mg/dL (or 240 μmol/L)
- *N*-terminal pro-brain natriuretic peptide (NT-pro-BNP) >986 pg/mL
- Left ventricular ejection fraction <45%

Some of the other predictors of relative poor outcome in chronic heart failure [9,10,41] are given below.

- High NYHA functional class
- Reduced left ventricular ejection fraction
- Third heart sound
- Increased pulmonary artery capillary wedge pressure
- Reduced cardiac index
- Diabetes mellitus
- Reduced sodium concentration
- Raised plasma catecholamine and natriuretic peptide concentrations

## 5. Management of Heart Failure

The major goals of treatment in heart failure are (1) to improve prognosis and reduce mortality and (2) to alleviate symptoms and reduce morbidity by reversing or slowing the cardiac and peripheral dysfunction. For in-hospital patients, in addition to the above goals, other goals of therapy are (1) to reduce the length of stay and subsequent readmission (2) to prevent organ system damage and (3) to appropriately manage the co-morbidities that may contribute to poor prognosis [42].

The 2013 American College of Cardiology/American Heart Association (ACC/AHA) updated guidelines [9], 2010 Heart Failure Society of America (HFSA) guidelines [12] and the 2008 European Society of Cardiology (ESC) [43] guidelines, with varying levels of evidence, recommend the following for different categories of HF patients.

### 5.1. In-Patient Management of HF

‘In-patient’ management of HF: It is advised to admit the patient in the telemetry bed or in ICU and the treatment is based on the following points.

- Monitor oxygen, whether PaO_2_ < 60% or SaO_2_ < 90%.
- Provide noninvasive positive pressure ventilation (NIPPV) in the few cases with respiratory distress for respiratory support to avoid subsequent intubation.
- Use the following pharmacological agents depending on the precipitating factors and symptoms/signs for congestion:
  - Diuretics (thiazides, loop diuretics and potassium sparing) (to reduce the edema by the reduction of blood volume and venous pressure) and salt restriction (to reduce fluid retention) in patients with current or previous heart failure symptoms and reduced left ventricular ejection fraction (LVEF) for symptomatic relief.
  - Angiotensin-converting enzyme inhibitors (ACEIs) or angiotensin receptor blockers (ARBs) for neuro-hormonal modification, vasodilatation and improvement in LVEF (substitute them with hydralazine and/or nitrates in patients unresponsive to ACEIs and ARBs).
  - Beta-adrenergic blockers for neuro-hormonal modification, improvement in symptoms and LVEF, survival benefit, arrhythmia prevention and control of ventricular rate.
  - Aldosterone antagonists, as an adjunct to other drugs for additive diuresis, heart failure symptom control, improved heart rate variability, decreased ventricular arrhythmias, reduction in cardiac workload, improved LVEF and an increase in survival.
  - Digoxin, which can lead to a small increase in cardiac output, improvement in heart failure symptoms and a decreased rate of heart failure hospitalizations.
  - Anticoagulants, if applicable, to decrease the risk of thromboembolism.
  - Inotropic agents to restore organ perfusion and reduce congestion in patients with heart failure with reduced ejection fraction, so as to increase in cardiac output and reduce neuro-humoral activation.
  - Some other agents have been described under clinical trial (Table 2).

In the case of refractory HF, ultrafiltration therapy is used for fluid reduction for patients that are not responsive to medical therapy [9]. In patients with NYHA Class III HF (and above) with the presentation of respiratory distress, symptomatic hypotension, impaired perfusion, worsening renal function and cardiogenic shock, invasive hemodynamic monitoring is recommended to guide therapy and improve outcome. The FDA approved the first permanently-implantable wireless hemodynamic monitoring system CardioMEMS Sensor (St. Jude Medical, St. Paul, MN, USA) for patients with NYHA Class III heart failure with a history of hospitalization for heart failure within the previous year based on an open label study, which showed a 30% reduction in hospitalization [44]. Several guidelines are published emphasizing the importance of ambulatory hemodynamic monitoring heart failure and the feasibility of a home monitoring system coupled with multidisciplinary and multi-level healthcare accessibility to improve health status and reduce HF hospitalizations [45,46].

#### Discharge Criteria for HF Patients

The patients of ADHF are ready for discharge when they meet the following criteria [9,10]:

1. Exacerbating factors have been addressed and are under control
2. Volume status has been optimized
3. Diuretic therapy has been successfully transitioned to oral medication, with discontinuation of IV vasodilator and inotropic therapy if required for at least 24 h
4. Oral therapy for chronic heart failure (HF), including angiotensin convertase enzyme inhibitors (ACEIs) and beta blockers (for patients with reduced LVEF), has been established with stable clinical status
5. Patient and family education completed, including clear discharge instructions
6. Left ventricular ejection fraction (LVEF) documented: echocardiography is the gold standard
7. Smoking cessation (if applicable) counseling initiated
8. Follow-up clinic visit scheduled within three days of discharge, usually for 7–10 days

For patients with advanced HF or recurrent admissions for HF, before discharge, the following are preferred [9,10]:

1. Oral medication regimen for heart failure has been established for 24 h
2. No intravenous vasodilator or inotropic agent for at least 24 h
3. Ambulation before discharge to evaluate the beneficial effect of therapy and restoration of functional capacity
4. Plans for post-discharge management to prevent readmission (scale present in home, visiting nurse or telephone follow-up generally no longer than three days after discharge)
5. Appropriate referral to a specialist for disease management of precipitant cause(s) if applicable

The discharge plan for the hospitalized patients should address the following issues [9,10]:

1. Medication reconciliation, written plans for dietary sodium restriction and recommended activity level
2. Follow-up by phone or clinic visit soon after discharge to reassess volume status
3. Medication and dietary adherence
4. Alcohol moderation and cessation of smoking
5. Monitoring of body weight, electrolytes and renal function
6. Consideration of referral for formal disease management

### 5.2. Out-Patient Management of HF [9,10]

1. Comprehensive education and counseling individualized to the patient’s disease and socio-economic and educational level
2. Education/promotion of self-care, including self-adjustment of diuretic therapy in appropriate patients with the help of a family member/caregiver
3. Early attention to signs and symptoms of fluid overload
4. Emphasis on behavioral strategies to increase adherence
5. Optimization of medical therapy
6. Vigilant follow-up after hospital discharge or after periods of instability
7. Increased access to providers or healthcare/social services
8. Assistance with social and financial concerns

## 6. Readmission

Readmission is defined as a subsequent hospital admission within 30 days following an original admission (or index stay). The overall 30-day readmission rate nation-wide (USA) is about 23%–26% [47]. Among Medicare patients hospitalized for HF from 2008–2010, 67.4% experienced a readmission and 35.8% died within one year of the index hospitalization. Several studies have been performed to determine the causes for the 30-day readmission [8,48,49,50], and some of the major causes include:

(1) Medication noncompliance (21%–66%)
(2) Smoking (60%)
(3) Sodium- and fluid-restricted diet noncompliance (30%–44%)
(4) Failure of documentation of discharge information and patient education (41%)
(5) Co-morbidities (21%–34%) (hypertension, diabetes mellitus, metabolic syndrome and atherosclerotic disease, anemia, depression)

In light of the repeated readmission of HF patients, the Affordable Care Act (ACA) of 2010 required HHS (Department of Health and Human Services) to establish a readmission reduction program. This program, effective 1 October 2012, was designed to provide incentives for hospitals to implement strategies to reduce the number of costly and unnecessary hospital readmissions, especially for diseases of Medicare diagnosis-related groups. Under this program, ACA implemented a financial penalty of up to three percent of their Medicare payments to hospitals for excessive readmissions for hospitals [51]. A few studies argue that the 30-day readmission measure failed to adjust for medical complexity, disability and socioeconomic status. Furthermore, hospitals in lower socioeconomic areas were found to be at a disadvantageous status and most likely to harbor increased risk for Medicare penalties. According to Rajaram et al., 2015, an estimated 58% of the national variation in hospital readmission rates were explained by the county socioeconomic factors [52]. The HRRP’s approach to calculating hospital penalties needs refinement to achieve the goal of reducing readmissions without unfairly penalizing hospitals [53]. At the same time, the Hospital Value-Based Purchasing (VBP) Program has been implemented as a Centers for Medicare and Medicaid Services (CMS) initiative that rewards acute-care hospitals with incentive payments for the quality of care they provide to people with Medicare. Several studies have determined the predictors of re-admissions [54,55], which include:

(1) Medical predictors: severity of orthopnea, renal dysfunction, hemodynamic instability, high levels of Pro-BNP, hyponatremia and presence of co-morbidities.
(2) Demographic predictors: male gender, advanced age, previous admission within six months, low median income, lack of psychosocial support, medication compliance and compliance to follow-up visits.

Various studies have implicated different strategies to bring reduction to the 30-day all-cause readmission rates [45,46,47,48,51,56,57,58,59,60]. These strategies include:

- Multidisciplinary HF clinics/centers (reduces all-cause readmission rates by 50%)
- Visiting nurse services and nurse specialist (reduces all-cause readmission rates by 37%)
- Physician-directed heart failure transitional care program (reduces all-cause readmission rates by 21%)
- Home tele-monitoring or structured phone calls (reduces all-cause readmission rates by 20%)
- Follow-up one-week post discharge (reduces all-cause readmission rates by 10%–15%)
- Transition care intervention home program (reduces all-cause readmission rates by 6%–12%)

These strategies have been further refined through the incorporation of the findings from the completed and ongoing clinical trials (e.g., PCDM-patient-centered disease management, REACH-HF-rehabilitation enablement in chronic heart failure [61,62] and Table 2).

## 7. Quality Improvement Strategies for HF

We have achieved great success in the optimization of pharmacological therapy along with the relative increase in the availability of better healthcare options. This has led to the reduction in the mortality in comparison to one seen in the 1970s [19]. On the contrary, this has led to rise in prevalence of HF and proportionate increase in the burden on the healthcare system, especially when associated with extended and frequent readmissions. The long-term goal of the treatment and management of HF is to avoid exacerbation of HF and to decrease the hospital readmission rate. The achievement of this goal encompasses an interdisciplinary approach involving patients and their physicians, nurses, family and care takers. Various reports have discussed the strategies to improve the overall quality of care of the patients of HF [11,60,63,64]. We have tried to summarize the crucial ones below.

1. Patient education:
  - patient education about HF and strategies for its treatment.
  - dietary counseling about sodium (2–3 gm/day; <2 gm/day may be considered in moderate to severe heart failure) and fluid restriction <2 L/day is considered when the fluid retention persists and when severe hyponatremia (serum Na <130 mEq/L) is present.
  - healthy lifestyle changes (high fiber diet with vegetables; regular exercise in a tolerable amount under monitoring of a cardiac rehabilitation program; consuming alcohol in moderation and no smoking); especially, recent studies have advocated the importance of exercise training to HF patients via improvement in the skeletal muscle O_2_ delivery, while simultaneously correcting mitochondrial and contractile efficiency. The localized muscle training has been shown to improve convective and diffusive O_2_ transport in HF and, hence, is useful for patients with minimal lung reserve capacity; several variables, such as exercise type, duration, frequency, intensity, etc., need to be taken into consideration to best benefit from such training [56,65,66].
  - efforts to improve patients’ compliance with medical regimens and interventions, such as phone calls, reminders and home nurse, to help patients remember to take the medications.
  - understand the alarming signs and symptoms, such shortness of breath, excessive fatigue, swelling of feet/ankle, etc. [11].
  - weight monitoring (weight daily and record: (i) get up in the morning; (ii) empty bladder; (iii) step on the scale and record) [9,11].
2. Arranging follow-up care: This includes assistance in scheduling the first follow-up appointment post-hospitalization along with re-enforcement of the importance of other follow-up visits. It also includes documentation of the date, time and location of the follow-up visit on the discharge plan, as well as sending reminders for subsequent appointments. One recent study has shown that it is possible to predict the readmission based on the response of the patients on the automated follow-up questionnaire [67].
3. Home tele-monitoring: This is a unique approach where the transmission of clinical parameters and symptoms of patients with HF at home to their healthcare provider, such as weight, blood pressure, heart rate, oxygen saturation, along with patients queries and questions regarding medications and symptoms and signs is conducted, thereby titrating the therapy based on the symptoms and signs. A few studies have shown that home tele-monitoring reduces mortality and hospitalizations, while in other studies, home tele-monitoring was found to be equivalent to telephone calls by a nurse [57].
4. Transition home program: This helps patients to have a safe transition to home or to another healthcare setting, such as a skilled nursing facility, and includes thorough patient and caregiver education, enhanced individualized assessment of post-discharge needs, patient-centered communication with caregivers and a standardized process for further management of HF along with follow-up visits with healthcare professionals [59,60,68].
5. Nurse assurance program: This program facilitates home service to follow-up on the patients with HF [58].
6. Specialized referral or health centers: This is designed to provide personalized care to HF patients with thorough assessment for heart transplantation needs. The referral to an HF program is shown to result in a decrease in the frequency of hospitalization of ≈50% [59,68].

**Figure 1:**
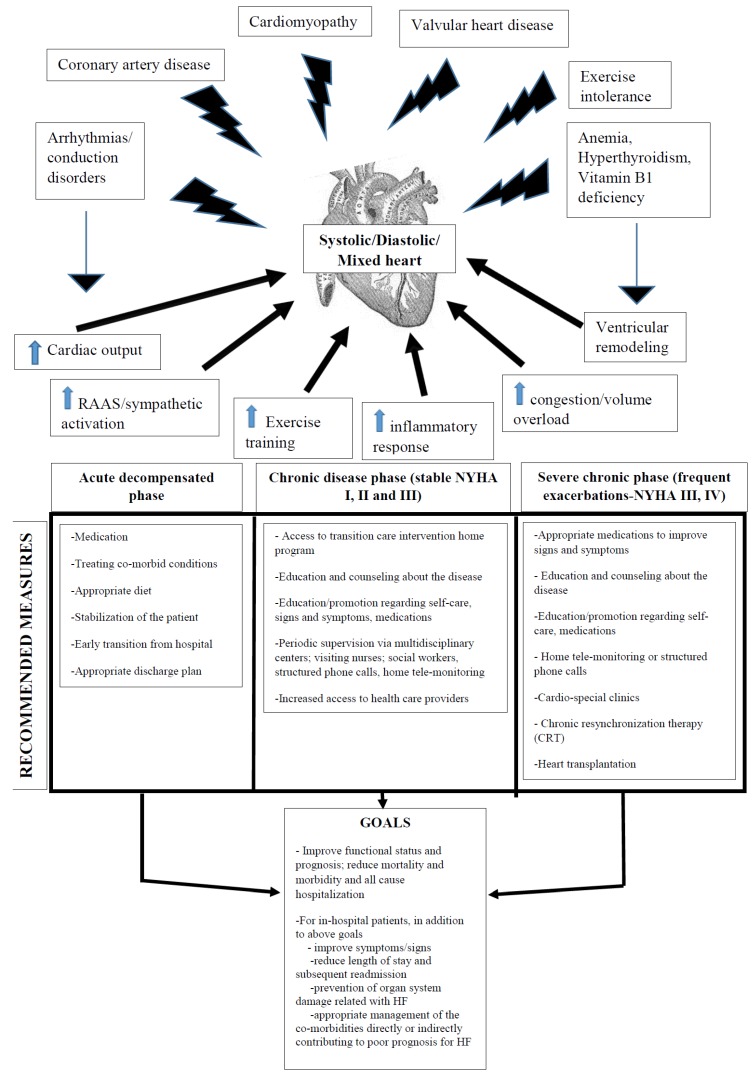
A schematic diagram showing the pathogenic mechanism for heart failure, as well as the important recommended measures so as to meet the goals of the heart failure treatment. NYHA, New York Heart Association.

The schematic diagram summarizing the causes and pathogenesis of HF along with an in-depth description of the management strategies based on the different phases of the HF in order to meet the recommended goals of the HF management is shown in Figure 1.

## 8. Standard and Novel Therapies for HF

### 8.1. Landmark Clinical Trials in the Management of HF

There have been numerous clinical trials all around the world as early as the 1990s CONSENSUS clinical trial, which determined the efficacy of diuretics for symptomatic HF. Soon, other clinical trials were designed to identify the best possible therapeutic agent to improve the clinical outcome of HF via pharmacological, non-pharmacological and novel treatment strategies. Table 2 summarizes the landmark clinical trials in the field of HF where HF was determined to be the primary problem without any other associated comorbidity or additional diagnosis. Recent clinical trials, such as SHIFT, EMPHASIS-HF and PARADIGM-HF, have focused more on advanced HF considering that current management of HF often fails to prevent the progression of HF to higher/advanced stages [69,70,71,72]. Such patients are also found to be benefited by ionotropic agents and even ultrafiltration procedures which relieve the congestion in resistant cases [73,74]. Many patients of HF require an intra-cardiac defibrillator (ICD) with or without chronic resynchronization therapy (CRT), which involves the implantation of a biventricular pacemaker (BVP) capable of stimulating both ventricles simultaneously so as to maintain the optimal cardiac output (CO) [75]; however, more trials are needed to understand the utility of ICD with or without CRT for HF [76]. More recent clinical trials have included stem cells and gene therapy in their regimen due to the self-renewal and differentiation of stem cells into myocytes. Several clinical trials involving cell therapy, especially with mesenchymal stem cells (MSCs), demonstrated that not only regeneration of the lost myocardium is possible, but also showed that the cell therapy can counteract the over-activation of inflammatory and immunological reactions after cardiac injury and, thus, improve the myocardial performance after the injury, attenuating adverse ventricular remodeling and decreasing myocardial fibrosis. Implantation of stem cells also improves the left ventricular ejection fraction and the overall quality of life; however, several conditions, such as area and mode of injection, source, type and number of cells and, more importantly, precise assessment of the end points are some of the factors that need to be optimized before these therapies can be routinely used for the treatment of HF [77]. In terms of gene therapy, overall progress has been slow, and relatively few clinical trials have been published so far for HF [78,79]. Gene therapy can be an excellent tool in medicine if progress can be made to precisely incorporate the appropriate target gene to reverse the pathological changes associated with failing heart. We advocate that instead of a single intervention, clinical trials with a combined approach comprised of pharmacological therapy, gene therapy and stem cell therapy at specific time intervals during the progression of the disease should be designed to inhibit or reverse the pathological processes causing the deterioration of the failing heart. Genetically-modified stem cells could be the next tool for the safe and effective application of gene therapy as explained in the next section.

### 8.2. Role of Cardiac Rejuvenation Therapy in the Management of HF

Current medical management for heart failure only alleviates symptoms, delays deterioration and prolongs life modestly. As the science has progressed by leaps and bounds, the idea of rejuvenation of the failing myocardium has begun to seem feasible when the accumulating evidence from preclinical studies demonstrated that rejuvenating the myocardium at the molecular and cellular level can be achieved by gene therapy and stem cell transplantation [111].

Stem cells are the population of cells that have self-renewal properties and the potential to generate daughter cells capable of differentiating into specific cell lineages [112]. Stem cells have shown promise to treat several human diseases due to their regenerative properties, and the idea of regeneration of myocardial damage or replacement of lost or damaged myocardial tissue by implanting stem cells has revolutionized the prospects in medicine. As far as heart failure (HF) is concerned, stem cells from both autologous and non-autologous sources are seen as feasible and efficient potential therapeutic agents. Several clinical trials using both autologous and allogenic stem cells have proven beneficial to patients of ischemic and non-ischemic heart failure in various clinical trials ([113,114,115] and references in Table 2). Stem cells can be isolated from various sources viz. human-derived myoblast, cardiosphere, mesenchymal, embryonic and menstrual blood.

The stem cell applications should be preferably undertaken in cases of acute injury. For example, the background pathophysiology is significantly different between chronic ischemic heart failure and acute myocardial infarction. This scenario is especially beneficial in acute myocardial infarction where the injured heart tissue secretes the inflammatory cytokines, which may even help in the homing of the infused stem cells to the injured tissue by the mechanism of chemotaxis. Thus, it will be easier for the stem cells to impact their beneficial effect, thus enhancing grafting and minimizing the degenerative remodeling, if the therapy is provided immediately after the myocardial injury. In hearts associated with acute myocardial infarction, the tissue is freshly injured and has not undergone remodeling which is often the case in chronic ischemic HF. Once cardiac remodeling has already taken place, the stem cells may not have a homing signal to graft into the infarcted site at the heart. Repeated injections of modified stem cells may also be an important aspect that has not been explored in the clinical application of stem cells. For patients of chronic ischemic heart disease, elective procedures to inject stem cells via epicardial or endocardial catheter have shown benefits. It has been seen that direct intramyocardial injections allow a greater myocardial retention of applied stem cells compared to that of intracoronary or systemic administration of stem cells [116].

Besides stem cell therapy, gene therapy equally holds promise in the field of HF. The success of gene therapy depends on the specific genes, types of vector and routes of application. For the successful application of gene therapy, the vectors should satisfy the criteria of efficient myocardium-specific transduction (specificity), high frequency of transduction (frequency) and long-term transgene expression (duration). The clinical outcomes of gene therapy have been limited due to obstacles like the development of neutralizing antibodies, cellular immunity against the viral vectors, immunity against the genetically-modified implanted cells and the low level of gene expression or transduction [117]. For example, the adenoviral vectors are not desirable due to their high inflammatory response. The adeno-associated viruses (AAV) are excellent vectors for cardiac gene therapy, not only because they satisfy these criteria, but also lack the immunogenic epitopes [118]. Cardiotrophic AAV serotypes have also been validated for cardiac-directed use, which makes AAV an attractive choice of vector. Lentiviruses present another serious alternative to AAV. Lentiviruses provide a high frequency of cardiac transduction and provide long-term expression; however, their use must be evaluated against the potential risk of insertional mutagenesis. Intravenous delivery of vectors may not be the best approach for cardiac gene therapy, because the sufficient amount of vector may not reach the myocardium. While selecting the route of delivery for cardiac gene therapy, direct intra-myocardial injection may be the choice of delivery to provide guaranteed localized transduction, as it can be delivered at the time of cardiac surgery [118,119].

The molecular targets of cardiac gene therapy can be well defined based on the participation of the gene in a specific function [120]. The angiogenic proteins, like vascular endothelial growth factor (VEGF) and fibroblast growth factor (FGF) help with improving perfusion by collateral vessel formation by increaseing angiogenesis. Such angiogenic gene therapy could be useful in the treatment of acute coronary syndrome and peripheral vascular disease [121]. The second group of genes important in cardiac gene therapy is comprised of proteins that affect the Ca^2+^ handling and myocardial contractility, such as adenylyl-cyclase 6 (AC6) and sarcoplasmic reticulum Ca^2+^ ATPase (SERCA2a), where SERCA2a is shown to be an inhibitor of ventricular remodeling [122]. Independent of the etiology of heart failure, the decreased SERCA2a level is partly responsible for heart failure. It also causes muscle relaxation by lowering the cytosolic calcium and restores the level of calcium in the sarcoplasmic reticulum, which is necessary for muscle contraction. AC6 triggers the conversion of ATP to cAMP, leading to phosphorylation of phospholamban (PLN). PLN is an inhibitor of SERCA2a, and its phosphorylation stops the inhibition of SERCA2a, making SERCA2a available for pumping the Ca^2+^ ions back to sarcoplasmic reticulum, reducing the cytoplasmic concentration of Ca^2+^ and allowing myofilament relaxation. Another important mention about cardiac gene therapy is Beta 2 adrenergic receptor therapy [123]. In animal models, the β-2 adrenergic receptor gene (β-2 AR) therapy has been shown to improve left ventricular systolic function and contractility response to isoproterenol. It has also been shown that overexpression of β-2 AR enhances VEGF production and increases endothelial cell proliferation and migration in animal models of ischemic limb [123]. In summary, a slow, but steady progress has been made in this field, and we hope to see gene therapy as a legitimate medical alternative in the physician’s arsenal in the coming decade [124].

## 9. Utilization and Medical Coding

In addition to having the knowledge of the pathophysiology of the HF and its management with the help of established and novel therapies, it is important for a physician to understand how to document the therapy so as to satisfy the reimbursement requirements. The utilization process ensures the appropriateness of the incurred healthcare costs by reviewing inpatient and outpatient services and comparing them against medical necessity guidelines. Usually the “clinical documentation improvement” (CDI) team facilitates the appropriate coding of the disease according to the guidelines and documents the codes in the International Classification of Diseases, Clinical Modification Version 10 (ICD-10-CM) mode. ICD-10 contains codes for human diseases, signs and symptoms, abnormal findings, social scenarios, external causes of injury or diseases and ‘diagnostic and procedure codes’ associated with inpatient, outpatient and physician office utilization in the United States [125]. Some of the ICD-10-CM for HF include I50—heart failure, I50.1—left ventricular failure, I50.2—systolic (congestive) heart failure, I50.3—diastolic (congestive) heart failure, I50.4—combined systolic (congestive) and diastolic (congestive) heart failure and I50.9—heart failure, unspecified [125].

The Centers for Medicare and Medicaid Services (CMS) implemented the National Correct Coding Initiative (NCCI) to promote correct coding methodologies and to control improper coding leading to inappropriate payment for the hospitalized patients. HF is classified under the Diagnosis-Related Group (DRG), which is a statistical system of classifying possible diagnoses into more than 20 major body systems and subdividing them into roughly 500 groups for the purpose of Medicare reimbursement. Factors used to determine the DRG payment amount include the involved diagnosis, as well as the hospital resources necessary to treat the condition. Based on the absence or presence of co-morbidity, DRGs are further sub-classified as ‘DRG with no complication/comorbidity’ (labeled as Non-CC); ‘DRGs with complication/comorbidity’ (CC) and ‘DRGs with major complication/comorbidity’ (MCC, where the presence of additional co-morbid conditions results in increased hospital resource utilization and impacts the MS-DRG payment to a major extent). The DRG codes for HF are categorized based on the severity, associated co-morbid conditions and reflect the level of utilization of hospital resources along with the payment reimbursement [126]. The following DRG codes are assigned for these categories: DRG 293 for HF without any comorbidity; DRG 292 for HF with comorbidity; and DRG 291 for HF with major comorbidity. Accordingly, ICM-10-CM has assigned the geometric length of stay (GLOS) for each DRG, which determines the average period of hospitalization required for improvement in the disease condition. GLOS determines the payment or reimbursement the hospital will receive for providing the care for the assigned period of stay. The GLOS for DRG 293, 292 and 291 is 2.6. LOS (length of stay) defines the actual period for which the patient remained in the hospital and is usually more than GLOS [126]. Each DRG has been assigned a weight, which is used to adjust for the fact that different types of patients consume different resources and have different costs. The diseases that require more resources have been assigned a higher weight than those that require fewer resources. Weights are updated annually to reflect the changes in medical practice patterns, the use of hospital resources, diagnostic and procedural definitions and DRG assignment criteria. Typically, reimbursement received by any hospital for a particular DRG is the hospital’s base rate determined by CMS multiplied by the DRG weight [126]. Physicians must be very specific when documenting the type of heart failure that has been diagnosed during hospital admission or a previous episode of care to get credit for a higher severity of illness and the corresponding payment increase [51]. For example, instead of documenting acute heart failure, based on the signs and symptoms, documentation should include the precise type of heart failure, such as acute systolic heart failure, or acute on chronic systolic heart failure, or acute diastolic heart failure, or possible chronic systolic heart failure, etc. Secondary diagnosis should also be as precise as possible. In addition, CPT codes, developed, maintained and copyrighted by the AMA (American Medical Association), are numbers assigned to ‘every task and service’ a medical practitioner provides to a patient, including medical, surgical and diagnostic services, and are used by insurance companies to determine the amount of reimbursement that a practitioner will receive for his/her service.

In summary, healthcare professionals should work with team members of the case-management department so as to provide precise documentation of the procedures needed for accurate prediction of the patient’s condition and diagnosis along with comorbid conditions, so as to receive the maximum reimbursement of payments.

## 10. Conclusions

Heart failure indeed is a complex disease and so far has been a major cause of morbidity and mortality in developing and developed countries. A standardized medical therapy has been successful in the early stages of HF. Advanced stages of HF require frequent hospitalization due to the presence of severe HF and or associated co-morbid conditions, which require strict implementation of an appropriately individualized multidisciplinary approach and quality measures to reduce re-admissions. While pharmacological management has a limited role in advanced cases of HF, novel therapeutic agents, such as regenerative and gene therapy, are in the developmental stages and need further refinement before their approval for the treatment of HF. Despite the appropriate measures, hospitalization in HF as a DRG has been a great challenge, especially since the adoption of the financial penalty program for excessive readmissions related to HF. In addition to the appropriate management of cases, healthcare professionals also need to provide precise and complete medical codes for procedures and diagnosis to help hospitals to receive the maximum reimbursement for the services provided to such patients.

## Figures and Tables

**Table 1:** The specific biomarkers expressed in heart failure (HF) patients as they correlate to the underlying mechanism of the pathogenesis for HF could be utilized for the diagnosis and prognosis of HF. Adapted from Ahmad et al., 2012 [30]. APO, apoptosis antigen; GDF, growth differentiation factor; ICAM, intercellular adhesion molecule; MMPs, matrix metalloproteinases; NGAL, neutrophil gelatinase-associated lipocalin; sST2, soluble ST2; TIMPs, matrix metalloproteinase tissue inhibitors.

Myocardial Stress	Myocardial Injury	Matrix and Cellular Remodeling	Inflammation	Oxidative Stress	Neuro-Hormones	Vascular System	Cardio-Renal Syndrome
Natriuretic	Cardiac troponins	Osteopontin	C-reactive protein	Oxidized LDL	Nor-epinephrine	Homocysteine	Creatinine
peptides	High sensitivity cardiac troponins	Galectin-3	sST2	Myeloperoxidase	Renin	Adhesion molecules	Cystatin C
Mid-regional	Myosin light-chain kinase 1	sST2	Tumor necrosis factor	Urinary biopyrrins	Angiotensin-II	ICAM, P-selectin	NGAL
Pro-adrenomedullin	Heart-type fatty acid binding protein	GDF-15	FAS (APO-1)	Urinary and plasma isoprostanes	Co-peptin	Endothelin	Trace protein
Neuregulin	Pentraxin 3	MMPs	GDF-15	Plasma malondialdehyde	Endothelin	Adiponectin	
sST2		TIMPs	Pentraxin 3			C-type natriuretic peptide	
		Collagen propeptides	Adipokines				
			cytokines				
			Procalcitonin				
			Osteoprotegerin				

**Table 2:** Pharmacological and Non-Pharmacological Clinical Trials for HF.

Clinical Trial Name	Drug Class	Drugs	Condition	Phase	No. of Patients	Date	Outcome	References
CONSENSUS	ACE inhibitors (ACEis)	Enalapril vs. placebo	Severe congestive heart failure	Double-blinded multi-center RCT	253	1987	ACEi improved symptoms, reduced HF progression in NYHA IV and mortality	[80]
SOLVD	ACE inhibitors (ACEis)	Enalapril vs. placebo	Heart failure with ejection fractions of 0.35 or less and on drugs other than an angiotensin-converting enzyme inhibitor	Double-blinded multi-center RCT	4228	1992	ACEi in an asymptomatic LV dysfunction reduced incidence and hospitalization for HF	[81]
RALES	Aldosterone antagonists	Spironolactone vs. placebo	CCF (NYHA III and IV)	Double-blinded multi-center RCT	1663	1999	Spironolactone reduced hospitalization (35%), mortality (30%) and symptoms in NYHA III/IV	[82]
CIBIS-II	Beta blockers	Bisoprolol vs. placebo	HF (NYHA Classes III–IV)	Double-blinded multi-center RCT	2647	1999	All-cause mortality hospitalizations and sudden cardiac death were reduced by 50%.	[83]
ValHeFT	Angiotensin receptor blockers (ARBs)	Valsartan vs. placebo	Heart failure (NYHA II–IV)	Multicenter, double-blinded, parallel-group, placebo-controlled RCT	5010	2001	Valsartan improved symptoms and mortality in NYHA II+; no benefit when added to ACEi	[84]
VMAC	Recombinant form of human B-type natriuretic peptide Vs nitrates	Intravenous nesiritide vs. nitroglycerin vs. placebo	Acute decompensated HF	Randomized, double-blind trial	489	2002	Nesiritide improved hemodynamic function as assessed by measuring reduced pulmonary capillary wedge pressure (PCWP)	[85]
COMET	Beta blockers	Carvedilol vs. metoprolol	Heart failure (EF < 35%; Stage II–IV)	Multicenter, double-blind, parallel-group, RCT	3029	2003	Carvedilol decreased all-cause mortality by 6% as compared to metoprolol	[86]
CHARM (includes CHARM added/alternative/preserved)	Angiotensin receptor blockers (ARBs)	Candesartan +/− ACEis vs. placebo	Heart failure (EF < 40%; Stage II or IV); (EF < 40% on ACEi for added); (EF < 40% intolerant of ACEi for alternative); EF > 40% for preserved	Double-blinded multi-center RCT	4576/2448 for added/2028 for alternative/30,233 for preserved	2003	Candesartan reduced death in HF; had added benefit in the presence of ACEi irrespective of ACEis dose; no benefit in preserved LV dysfunction	[87,88,89]
EVEREST	Vasopressin antagonists	Tolvaptan vs. placebo	Decompensated HF	Multi-center, double-blind, parallel-group, randomized controlled trial	4133	2007	Significant benefit on dyspnea, edema, body weight and serum sodium, but no improvement in cardio-vascular mortality or HF hospitalization	[90]
VERITAS	Endothelin receptor antagonist	Intravenous tezosentan vs. placebo	Acute HF	Randomized, double-blind trial	1435	2007	Tezosentan failed to improve symptoms or clinical outcomes in patients with acute heart failure	[91]
CORONA	Statin	Rosuvastatin vs. placebo	Congestive Cardiac Failure (CCF) (EF < 40%, NYHA II)	Multicenter, double-blind, randomized placebo-controlled trial	5011	2007	Rosuvastatin in statin-naive CCF patients reduced admissions, but not mortality	[92]
ACCLAIM	Device-based non-specific immuno-modulation therapy (IMT)	Celecade vs. placebo	NYHA II–IV HF	Double-blind, placebo-controlled study	2426	2008	Failed to demonstrate reduction in hospitalization or mortality, but proposed to be beneficial for the early stages of HF	[93]
SHIFT	Specific inhibitor of current in the sinoatrial node	Ivabradine vs. placebo	HF with LVEF 35% or lower with heart rate >70 in sinus rhythm	Double-blinded multi-center RCT	6558	2010	Ivabradine reduced CCF admissions and deaths, especially those with higher HR	[69]
EMPHASIS-HF	Aldosterone antagonists	Eplerenone vs. placebo	CCF (NYHA II and EF < 35%)	Double-blinded multi-center RCT	2737	2011	Eplerenone reduced mortality by 7% and symptoms in NYHA II	[70]
ASCEND-HF	Recombinant form of human B-type natriuretic peptide	Nesiritide infusion vs. placebo	HF	Double-blinded multi-center RCT	7141	2011	Improved the symptom of dyspnea, but no change in mortality	[72]
RELAX	cGMP-specific phosphodiesterase type 5 inhibitor	Sildenafil vs. placebo	Diastolic HF with NYHA II–III (LVEF > 50%)	Double-blinded multi-center RCT	216	2012	No improvement in health outcomes and exercise ability	[94]
ASTRONAUT	Renin inhibitor	Aliskiren vs. placebo	Decompensated HF	Multicenter, double-blind, randomized placebo-controlled trial	1639	2013	No additional benefit from the drug to standard therapy	[95]
ATOMIC-AHF	Cardiac-specific myosin activator	Omecamtiv mecarbil vs. placebo	ADHF with LVEF ≤ 40%	Multicenter, double-blind, randomized placebo-controlled trial	614	2013	Safe, but no change in the dyspnea symptoms	[96]
RELAX-AHF	Vasoactive peptide hormone	Serelaxin, recombinant human relaxin-2 vs. placebo	Acute HF	Randomized, placebo-controlled trial	1161	2013	Dyspnea relief and other symptoms of HF, but had no effect on hospital readmissions	[97]
PARADIGM-HF	Combination of ARB, valsartan and a neprilysin inhibitor prodrug sacubitril	Valsartan/sacubitril (LCZ696) vs. enalapril	NYHA functional Class II–IV (HFrEF and HFpEF)	Randomized study	8442	2014	Significant reductions in cardiovascular and all-cause mortality, as well as heart failure hospitalization	[72,98]
SOCRATES, including SOCRATES-REDUCED for LVEF ≤ 45 SOCRATES-PRESERVED for LVEF ≥ 45	Oral cyclic guanosine monophosphate (cGMP) stimulator	Oral (cGMP) stimulator vericiguat (BAY 1021189) vs. placebo	HF with LVEF ≥ 45 and ≤ 45	Double-blinded multi-center RCT	456	2014	Study completed, results awaited	[99]
NCT01919177	Inorganic nitrates	Beet root vs. placebo	Heart failure with normal ejection fraction	randomized, double-blind,	17	2015	Increased exercise capacity by increasing exercise vasodilatory and cardiac output reserves	[3]
**Defibrillator-based clinical trials**								
SCD-HeFT	ICD vs. drug	ICD vs. amiodarone vs. placebo	CCF (NYHA II/III; LVEF < 35)	Double-blinded multi-center RCT	2521	2005	ICD significantly increased survival by 23%; amiodarone had no effect	[100]
MADIT-CRT	CRT	CRT with and without ICD	HF (NYHA I–II; EF < 30%; QRS > 130 ms)	Double-blinded multi-center RCT	1820	2009	CRT (added to ICD) slows the progression of heart failure in high-risk (QRS ≥ 130 ms, EF ≤ 3 0%), mildly symptomatic patients (NYHA I/II)	[75]
PARTNERS HF	HF device	Combined heart failure (HF) device guided diagnostic data to predict clinical deterioration of HF	CRT implantable cardioverter-defibrillators in HF patients	Observational study	1024	2010	Identifies patients at a higher risk of HF hospitalizations	[101]
**Stem cell-based clinical trials**								
TOPCARE-CHD	Bone marrow-derived mononuclear cells	intracoronary injection of functional BMMC vs. placebo	Ischemic HF	Single-center study randomized	121	2007	Improved cardiac function and suppression of NT-proANP and proBNP with BMMC, especially with cells with high functional capacity determined with the colony forming unit assay	[102]
SCIPIO	Cardiac stem cells	Intracoronary injection of in vitro expanded c-Kit+ CSC from myocardium vs. placebo	Ischemic HF with LVEF < 40%	Single-center study	18	2011	Significant improvement in myocardial performance, scar tissue reduction and LV systolic function	[103]
TAC-HFT	MSCs and BMMCs	Trans-endocardial injection of culture-expanded MSCs vs. whole BMMC vs. placebo	Ischemic cardio-myopathy with LVEF < 50%	Randomized, blinded, placebo-controlled study	65	2011	MSCs and BMMC were safe, but MSCs better for scar reduction and improved myocardial function than BMMCs	[104]
FOCUS-CCTRN	Bone marrow-derived mononuclear cells	Trans-endocardial injection of BMMC vs. placebo	Ischemic HF/NYHA II–III with LVHF < 45%	Randomized double-blind, placebo-controlled trial	153	2012	Failed to improve LVESV, maximal oxygen consumption or reversibility on SPECT	[105]
POSEIDON	Mesenchymal stem cells	Allogenic vs. autologous trans-endocardial injection of MSCs	Chronic ischemic left ventricular dysfunction with LVHF < 50%	Single-center study	31	2012	Both allo- and auto-MSCs were safe, reduced infarct size and improved ventricular remodeling	[106]
CADUCEUS	Cardiosphere-derived cells	Intracoronary administration of autologous CDCs vs. placebo	Ischemic HF, NYHF I with LVEF between 25% and 45%	Single-center study	17	2012	Safe and decreased scar size, increased viable myocardium and improved regional function of infarcted myocardium, but no significant improvement in EF	[107]
NOGA-DCM	Bone marrow-derived CD34+ cells	Trans-endocardial CD34+ vs. placebo	Non-ischemic cardiomyopathy with NYHA III and LVHF < 40%	Single-center study randomized	33	2014	Improved left ventricular function, decreased *N*-terminal pro-BNP and better exercise capacity with infusion of a high number of cells	[108]
PROMETHEUS	Mesenchymal stem cells	Intra-myocardial injection of autologous MSCs	Chronic ischemic cardiomyopathy undergoing CABG	Single-center study	6	2014	Scar reduction, improvement in myocardial perfusion, regional function and LVEF in patients undergoing CABG	[109]
CHART-1	Cardiopoietic stem cells	bone marrow-derived and lineage-directed autologous cardiopoietic stem cells	Ischemic HF	Randomized, sham-controlled multicenter study	240	2015	Under progress	[110]
**Gene therapy-based clinical trials**								
CUPID-Phase I	Gene therapy	Antegrade epicardial coronary artery infusion of gene SERCA2a via an adeno-associated viral (AAV) vector	Advanced HF-NYHF III/IV (LVEF ≤ 30%)	Single-center study	9	2008	Safe and improvement in various parameters, such as exercise tolerance, LVEF, reduction of BNP levels	[78]
CUPID-Phase II	Gene therapy	Intracoronary adeno-associated virus type 1/sarcoplasmic reticulum Ca^2+^-ATPase vs. placebo	Advanced HF-NYHF III/IV (LVEF ≤ 30%)	Randomized, double-blind, placebo-controlled	39	2011	Improvement in various parameters, such as exercise tolerance, LVEF, reduction of BNP levels	[79]

## References

[1] Dassanayaka S., Jones S.P. (2015). Recent Developments in Heart Failure. Circ. Res..

[2] Ohtani T., Mohammed S.F., Yamamoto K. (2012). Diastolic stiffness as assessed by diastolic wall strain is associated with adverse remodeling and poor outcomes in heart failure with preserved ejection fraction. Eur. Heart J..

[3] Zamani P., Rawat D., Shiva-Kumar P., Geraci S., Bhuva R., Konda P., Doulias P.T., Ischiropoulos H., Townsend R.R., Margulies K.B. (2015). Effect of inorganic nitrate on exercise capacity in heart failure with preserved ejection fraction. Circulation.

[4] Glean A.A., Ferguson S.K., Holdsworth C.T., Colburn T.D., Wright J.L., Fees A.J., Hageman K.S., Poole D.C., Musch T.I. (2015). Effects of nitrite infusion on skeletal muscle vascular control during exercise in rats with chronic heart failure. Am. J. Physiol. Heart Circ. Physiol..

[5] Maeder M.T., Thompson B.R., Brunner-La Rocca H.-P., Kaye D.M. (2010). Hemodynamic basis of exercise limitation in patients with heart failure and normal ejection fraction. J. Am. Coll. Cardiol..

[6] Bhella P.S., Prasad A., Heinicke K., Hastings J.L., Arbab-Zadeh A., Adams-Huet B., Pacini E.L., Shibata S., Palmer M.D., Newcomer B.R. (2011). Abnormal haemodynamic response to exercise in heart failure with preserved ejection fraction. Eur. J. Heart Fail..

[7] Angadi S.S., Mookadam F., Lee C.D., Tucker W.J., Haykowsky M.J., Gaesser G.A. (2015). High-intensity interval training vs. moderate-intensity continuous exercise training in heart failure with preserved ejection fraction: A pilot study. J. Appl. Physiol. (1985).

[8] Paulus W.J., Tschöpe C. (2013). A novel paradigm for heart failure with preserved ejection fraction: Comorbidities drive myocardial dysfunction and remodeling through coronary microvascular endothelial inflammation. J. Am. Coll. Cardiol..

[9] Yancy C.W., Jessup M., Bozkurt B., Butler J., Casey D.E., Drazner M.H., Fonarow G.C., Geraci S.A., Horwich T., Januzzi J.L. (2013). 2013 ACCF/AHA guideline for the management of heart failure: A report of the American College of Cardiology Foundation/American Heart Association Task Force on Practice Guidelines. J. Am. Coll. Cardiol..

[10] Watson R.D., Gibbs C.R., Lip G.Y. (2000). ABC of heart failure. Clinical features and complications. BMJ Br. Med. J..

[11] Lindenfeld J., Albert N.M., Boehmer J.P., Collins S.P., Ezekowitz J.A., Givertz M.M., Katz S.D., Klapholz M., Moser D.K., Rogers J.G. (2010). HFSA 2010 Comprehensive Heart Failure Practice Guideline. J. Card. Fail..

[12] Marti C.N., Georgiopoulou V.V., Kalogeropoulos A.P. (2013). Acute heart failure: Patient characteristics and pathophysiology. Curr. Heart Fail. Rep..

[13] Poole D.C., Hirai D.M., Copp S.W., Musch T.I. (2012). Muscle oxygen transport and utilization in heart failure: Implications for exercise (in)tolerance. Am. J. Physiol. Heart Circ. Physiol..

[14] Heidenreich P.A., Albert N.M., Allen L.A., Bluemke D.A., Butler J., Fonarow G.C., Ikonomidis J.S., Khavjou O., Konstam M.A., Maddox T.M. (2013). Forecasting the impact of heart failure in the United States: A policy statement from the American Heart Association. Circ. Heart Fail..

[15] Dunlay S.M., Shah N.D., Shi Q., Morlan B., VanHouten H., Long K.H., Roger V.L. (2011). Lifetime costs of medical care after heart failure diagnosis. Circ. Cardiovasc. Qual. Outcomes.

[16] Askoxylakis V., Thieke C., Pleger S.T. (2010). Long-term survival of cancer patients compared to heart failure and stroke: A systematic review. BMC Cancer.

[17] Krumholz H.M., Lin Z., Keenan P.S., Chen J., Ross J.S., Drye E.E., Bernheim S.M., Wang Y., Bradley E.H., Han L.F. (2013). Normand SL. Relationship between hospital readmission and mortality rates for patients hospitalized with acute myocardial infarction, heart failure, or pneumonia. JAMA J. Am. Med. Assoc..

[18] Ni H., Xu J. (2015). Recent trends in heart failure-related mortality: United States, 2000–2014. NCHS Data Brief, 231.

[19] Roger V.L. (2013). Epidemiology of heart failure. Circ. Res..

[20] Anker S.D., von Haehling S. (2004). Inflammatory mediators in chronic heart failure: An overview. Heart.

[21] Hofmann U., Frantz S. (2013). How can we cure a heart “in flame”? A translational view on inflammation in heart failure. Basic Res. Cardiol..

[22] Oikonomou E., Tousoulis D., Siasos G., Zaromitidou M., Papavassiliou A.G., Stefanadis C. (2011). The role of inflammation in heart failure: New therapeutic approaches. Hell. J. Cardiol..

[23] Tang W.H., Wang Z., Fan Y., Levison B., Hazen J.E., Donahue L.M., Wu Y., Hazen S.L. (2014). Prognostic value of elevated levels of intestinal microbe-generated metabolite trimethylamine-*N*-oxide in patients with heart failure: Refining the gut hypothesis. J. Am. Coll. Cardiol..

[24] Nagatomo Y., Tang W.H. (2015). Intersections between Microbiome and Heart Failure: Revisiting the Gut Hypothesis. J. Card. Fail..

[25] Maries L., Manitiu I. (2013). Diagnostic and prognostic values of B-type natriuretic peptides (BNP) and *N*-terminal fragment brain natriuretic peptides (NT-pro-BNP). Cardiovasc. J. Afr..

[26] Pfister R., Scholz M., Wielckens K., Erdmann E., Schneider C.A. (2004). Use of NT-proBNP in routine testing and comparison to BNP. Eur. J. Heart Fail..

[27] Simons J.E., Don-Wauchope A.C. (2016). Evaluation of natriuretic peptide recommendations in heart failure clinical practice guidelines. Clin. Biochem..

[28] Maisel A., Hollander J.E., Guss D., McCullough P., Nowak R., Green G., Saltzberg M., Ellison S.R., Bhalla M.A., Bhalla V. (2004). Primary results of the Rapid Emergency Department Heart Failure Outpatient Trial (REDHOT). A multicenter study of B-type natriuretic peptide levels, emergency department decision making, and outcomes in patients presenting with shortness of breath. J. Am. Coll. Cardiol..

[29] Murtagh G., Canniffe C., Mahgoub M., Blake L., McCarroll N., Crowley V., Bennett K., Silke B. (2009). Introduction of an NT-proBNP assay to an acute admission unit—A 2-year audit. Eur. J. Intern. Med..

[30] Ahmad T., Fiuzat M., Felker G.M., O’Connor C. (2012). Novel biomarkers in chronic heart failure. Nat. Rev. Cardiol..

[31] Gaggin H.K., Januzzi J.L. (2013). Biomarkers and diagnostics in heart failure. Biochim. Biophys. Acta.

[32] van Kimmenade R.R., Januzzi J.L. (2012). Emerging biomarkers in heart failure. Clin. Chem..

[33] Paterson I., Mielniczuk L.M., O’Meara E., So A., White J.A. (2013). Imaging heart failure: Current and future applications. Can. J. Cardiol..

[34] Morbach C., Lin B.A., Sugeng L. (2014). Clinical application of three-dimensional echocardiography. Prog. Cardiovasc. Dis..

[35] Butler J. (2007). The emerging role of multi-detector computed tomography in heart failure. J. Card. Fail..

[36] Upadhya B., Haykowsky M.J., Eggebeen J., Kitzman D.W. (2015). Exercise intolerance in heart failure with preserved ejection fraction: More than a heart problem. J. Geriatr. Cardiol..

[37] Fonarow G.C., Yancy C.W., Heywood J.T., ADHERE Scientific Advisory Committee, Study Group, Investigators (2005). Adherence to heart failure quality-of-care indicators in US hospitals: Analysis of the ADHERE Registry. Arch. Intern. Med..

[38] Januzzi J.L., Sakhuja R., O’donoghue M., Baggish A.L., Anwaruddin S., Chae C.U., Cameron R., Krauser D.G., Tung R., Camargo C.A. (2006). Utility of amino-terminal pro-brain natriuretic peptide testing for prediction of 1-year mortality in patients with dyspnea treated in the emergency department. Arch. Intern. Med..

[39] West R., Liang L., Fonarow G.C., Kociol R., Mills R.M., O’Connor C.M., Hernandez A.F. (2011). Characterization of heart failure patients with preserved ejection fraction: A comparison between ADHERE-US registry and ADHERE-International registry. Eur. J. Heart Fail..

[40] Ouwerkerk W., Voors A.A., Zwinderman A.H. (2014). Factors influencing the predictive power of models for predicting mortality and/or heart failure hospitalization in patients with heart failure. JACC Heart Fail..

[41] Pocock S.J., Wang D., Pfeffer M.A., Yusuf S., McMurray J.J., Swedberg K.B., Ostergren J., Michelson E.L., Pieper K.S., Granger C.B. (2006). Predictors of mortality and morbidity in patients with chronic heart failure. Eur. Heart J..

[42] Tamargo J., López-Sendón J. (2011). Novel therapeutic targets for the treatment of heart failure. Nat. Rev. Drug Discov..

[43] Dickstein K., Cohen-Solal A., Filippatos G., McMurray J.J., Ponikowski P., Poole-Wilson P.A., Strömberg A., van Veldhuisen D.J., Atar D., Hoes A.W. (2008). ESC guidelines for the diagnosis and treatment of acute and chronic heart failure 2008: The Task Force for the diagnosis and treatment of acute and chronic heart failure 2008 of the European Society of Cardiology. Developed in collaboration with the Heart Failure Association of the ESC (HFA) and endorsed by the European Society of Intensive Care Medicine (ESICM). Eur. J. Heart Fail..

[44] Abraham W.T., Adamson P.B., Bourge R.C., Aaron M.F., Costanzo M.R., Stevenson L.W., Strickland W., Neelagaru S., Raval N., Krueger S. (2011). Wireless pulmonary artery haemodynamic monitoring in chronic heart failure: A randomised controlled trial. Lancet.

[45] Bui A.L., Fonarow G.C. (2012). Home monitoring for heart failure management. J. Am. Coll. Cardiol..

[46] Guidi G., Pollonini L., Dacso C.C., Iadanza E. (2015). A multi-layer monitoring system for clinical management of Congestive Heart Failure. BMC Med. Inform. Decis. Mak..

[47] Dharmarajan K., Hsieh A.F., Lin Z., Bueno H., Ross J.S., Horwitz L.I., Barreto-Filho J.A., Kim N., Bernheim S.M., Suter L.G. (2013). Diagnoses and Timing of 30-Day Readmissions After Hospitalization for Heart Failure, Acute Myocardial Infarction, or Pneumonia. JAMA J. Am. Med. Assoc..

[48] VanSuch M., Naessens J.M., Stroebel R.J., Huddleston J.M., Williams A.R. (2006). Effect of discharge instructions on readmission of hospitalised patients with heart failure: Do all of the Joint Commission on Accreditation of Healthcare Organizations heart failure core measures reflect better care?. Qual. Saf. Health Care.

[49] Donzé J., Lipsitz S., Bates D.W., Schnipper J.L. (2013). Causes and patterns of readmissions in patients with common comorbidities: Retrospective cohort study. BMJ.

[50] Desai A.S. (2012). The three-phase terrain of heart failure readmissions. Circ. Heart Fail..

[51] Center for Medicare and Medicaid Services. www.CMS.gov.

[52] Rajaram R., Chung J.W., Kinnier C.V., Barnard C., Mohanty S., Pavey E.S., McHugh M.C., Bilimoria K.Y. (2015). Hospital Characteristics Associated With Penalties in the Centers for Medicare & Medicaid Services Hospital-Acquired Condition Reduction Program. JAMA J. Am. Med. Assoc..

[53] Barlas S. (2013). Hospitals Prepare for Medicare Cuts: The Need to Reduce the Federal Deficit Puts Seniors and Providers on Notice. Pharm. Ther..

[54] Desai A.S., Stevenson L.W. (2012). Rehospitalization for heart failure: Predict or prevent?. Circulation.

[55] Hernandez M.B., Schwartz R.S., Asher C.R., Navas E.V., Totfalusi V., Buitrago I., Lahoti A., Novaro G.M. (2013). Predictors of 30-day readmission in patients hospitalized with decompensated heart failure. Clin. Cardiol..

[56] Jolly K., Taylor R.S., Lip G.Y., Davies M., Davis R., Mant J., Singh S., Greenfield S., Ingram J., Stubley J. (2009). A randomized trial of the addition of home-based exercise to specialist heart failure nurse care: The Birmingham Rehabilitation Uptake Maximisation study for patients with Congestive Heart Failure (BRUM-HF) study. Eur. J. Heart Fail..

[57] Inglis S.C., Clark R.A., McAlister F.A., Ball J., Lewinter C., Cullington D., Stewart S., Cleland J.G. (2010). Structured telephone support or telemonitoring programmes for patients with chronic heart failure. Cochrane Database Syst. Rev..

[58] Butler J., Kalogeropoulos A. (2012). Hospital strategies to reduce heart failure readmissions: Where is the evidence?. J. Am. Coll. Cardiol..

[59] Ota K.S., Beutler D.S., Gerkin R.D., Weiss J.L., Loli A.I. (2013). Physician-directed heart failure transitional care program: A retrospective case review. J. Clin. Med. Res..

[60] Feltner C., Jones C.D., Cené C.W., Zheng Z.J., Sueta C.A., Coker-Schwimmer E.J., Arvanitis M., Lohr K.N., Middleton J.C., Jonas D.E. (2014). Transitional care interventions to prevent readmissions for persons with heart failure: A systematic review and meta-analysis. Ann. Intern. Med..

[61] Bekelman D.B., Plomondon M.E., Carey E.P., Sullivan M.D., Nelson K.M., Hattler B., McBryde C.F., Lehmann K.G., Gianola K., Heidenreich P.A. (2015). Primary Results of the Patient-Centered Disease Management (PCDM) for Heart Failure Study: A Randomized Clinical Trial. JAMA Intern. Med..

[62] Taylor R.S., Hayward C., Eyre V., Austin J., Davies R., Doherty P., Jolly K., Wingham J., Van Lingen R., Abraham C. (2015). Clinical effectiveness and cost-effectiveness of the Rehabilitation Enablement in Chronic Heart Failure (REACH-HF) facilitated self-care rehabilitation intervention in heart failure patients and caregivers: Rationale and protocol for a multicentre randomised controlled trial. BMJ Open.

[63] Giamouzis G., Kalogeropoulos A., Georgiopoulou V., Laskar S., Smith A.L., Dunbar S., Triposkiadis F., Butler J. (2011). Hospitalization epidemic in patients with heart failure: Risk factors, risk prediction, knowledge gaps, and future directions. J. Card. Fail..

[64] Scalvini S., Giordano A. (2013). Heart failure. Optimal postdischarge management of chronic HF. Nat. Rev. Cardiol..

[65] Fleg J.L., Cooper L.S., Borlaug B.A., Haykowsky M.J., Kraus W.E., Levine B.D., Pfeffer M.A., Piña I.L., Poole D.C., Reeves G.R. (2015). Exercise training as therapy for heart failure: Current status and future directions. Circ. Heart Fail..

[66] Hirai D.M., Musch T.I., Poole D.C. (2015). Exercise training in chronic heart failure: Improving skeletal muscle O2 transport and utilization. Am. J. Physiol. Heart Circ. Physiol..

[67] Inouye S., Bouras V., Shouldis E., Johnstone A., Silverzweig Z., Kosuri P. (2015). Predicting readmission of heart failure patients using automated follow-up calls. BMC Med. Inform. Decis. Mak..

[68] Thomas R., Huntley A., Mann M., Huws D., Paranjothy S., Elwyn G., Purdy S. (2013). Specialist clinics for reducing emergency admissions in patients with heart failure: A systematic review and meta-analysis of randomised controlled trials. Heart.

[69] Swedberg K., Komajda M., Böhm M., Borer J.S., Ford I., Dubost-Brama A., Lerebours G., Tavazzi L., SHIFT Investigators (2010). Ivabradine and outcomes in chronic heart failure (SHIFT): A randomised placebo-controlled study. Lancet.

[70] Zannad F., McMurray J.J., Krum H., van Veldhuisen D.J., Swedberg K., Shi H., Vincent J., Pocock S.J., Pitt B., EMPHASIS-HF Study Group (2011). Eplerenone in patients with systolic heart failure and mild symptoms. N. Engl. J. Med..

[71] O’Connor C.M., Starling R.C., Hernandez A.F., Armstrong P.W., Dickstein K., Hasselblad V., Heizer G.M., Komajda M., Massie B.M., McMurray J.J. (2011). Effect of nesiritide in patients with acute decompensated heart failure. N. Engl. J. Med..

[72] McMurray J.J., Packer M., Desai A.S., Gong J., Lefkowitz M., Rizkala A.R., Rouleau J.L., Shi V.C., Solomon S.D., Swedberg K. (2014). Baseline characteristics and treatment of patients in prospective comparison of ARNI with ACEI to determine impact on global mortality and morbidity in heart failure trial (PARADIGM-HF). Eur. J. Heart Fail..

[73] Goldhaber J.I., Hamilton M.A. (2010). Role of inotropic agents in the treatment of heart failure. Circulation.

[74] Krishnamoorthy A., Felker G.M. (2014). Fluid removal in acute heart failure: Diuretics *versus* devices. Curr. Opin. Crit. Care.

[75] Moss A.J., Hall W.J., Cannom D.S., Klein H., Brown M.W., Daubert J.P., Estes N.A., Foster E., Greenberg H., Higgins S.L. (2009). Cardiac-resynchronization therapy for the prevention of heart-failure events. N. Engl. J. Med..

[76] Cleland J.G., Mareev Y. (2015). CRT for Heart Failure and ESRD: More Trials or More Thought Needed?. J. Am. Coll. Cardiol..

[77] Huang Y., Mai L., Cai X., Hu Y., Mai W. (2016). Stem cell therapy for heart disease-Meta-analysis may be misleading. Int. J. Cardiol..

[78] Jaski B.E., Jessup M.L., Mancini D.M., Cappola T.P., Pauly D.F., Greenberg B., Borow K., Dittrich H., Zsebo K.M., Hajjar R.J. (2009). Calcium upregulation by percutaneous administration of gene therapy in cardiac disease (CUPID Trial), a first-in-human phase 1/2 clinical trial. J. Card. Fail..

[79] Jessup M., Greenberg B., Mancini D., Cappola T., Pauly D.F., Jaski B., Yaroshinsky A., Zsebo K.M., Dittrich H., Hajjar R.J. (2011). Calcium Upregulation by Percutaneous Administration of Gene Therapy in Cardiac Disease (CUPID): A phase 2 trial of intracoronary gene therapy of sarcoplasmic reticulum Ca^2+^-ATPase in patients with advanced heart failure. Circulation.

[80] The Consensus Trial Study Group (1987). Effects of enalapril on mortality in severe congestive heart failure. Results of the Cooperative North Scandinavian Enalapril Survival Study (CONSENSUS). N. Engl. J. Med..

[81] The SOLVD Investigators (1992). Effect of enalapril on mortality and the development of heart failure in asymptomatic patients with reduced left ventricular ejection fractions. N. Engl. J. Med..

[82] Pitt B., Zannad F., Remme W.J., Cody R., Castaigne A., Perez A., Palensky J., Wittes J. (1999). The effect of spironolactone on morbidity and mortality in patients with severe heart failure. Randomized Aldactone Evaluation Study Investigators. N. Engl. J. Med..

[83] (1999). The Cardiac Insufficiency Bisoprolol Study II (CIBIS-II): A randomised trial. Lancet.

[84] Cohn J.N., Tognoni G., Valsartan Heart Failure Trial Investigators (2001). A randomized trial of the angiotensin-receptor blocker valsartan in chronic heart failure. N. Engl. J. Med..

[85] Publication Committee for the VMAC Investigators (Vasodilatation in the Management of Acute HF) (2002). Intravenous nesiritide vs. nitroglycerin for treatment of decompensated congestive heart failure: A randomized controlled trial. JAMA J. Am. Med. Assoc..

[86] Poole-Wilson P.A., Swedberg K., Cleland J.G., Di Lenarda A., Hanrath P., Komajda M., Lubsen J., Lutiger B., Metra M., Remme W.J. (2003). Carvedilol Or Metoprolol European Trial Investigators. Comparison of carvedilol and metoprolol on clinical outcomes in patients with chronic heart failure in the Carvedilol or Metoprolol European Trial (COMET): Randomised controlled trial. Lancet.

[87] McMurray J.J., Ostergren J., Swedberg K., Granger C.B., Held P., Michelson E.L., Olofsson B., Yusuf S., Pfeffer M.A., CHARM Investigators and Committees (2003). Effects of candesartan in patients with chronic heart failure and reduced left-ventricular systolic function taking angiotensin-converting-enzyme inhibitors: The CHARM-Added trial. Lancet.

[88] Granger C.B., McMurray J.J., Yusuf S., Held P., Michelson E.L., Olofsson B., Ostergren J., Pfeffer M.A., Swedberg K., CHARM Investigators and Committees (2003). Effects of candesartan in patients with chronic heart failure and reduced left-ventricular systolic function intolerant to angiotensin-converting-enzyme inhibitors: The CHARM-Alternative trial. Lancet.

[89] Yusuf S., Pfeffer M.A., Swedberg K., Granger C.B., Held P., McMurray J.J., Michelson E.L., Olofsson B., Ostergren J., CHARM Investigators and Committees (2003). Effects of candesartan in patients with chronic heart failure and preserved left-ventricular ejection fraction: The CHARM-Preserved Trial. Lancet.

[90] Konstam M.A., Gheorghiade M., Burnett J.C., Grinfeld L., Maggioni A.P., Swedberg K., Udelson J.E., Zannad F., Cook T., Ouyang J. (2007). Efficacy of Vasopressin Antagonism in Heart Failure Outcome Study With Tolvaptan (EVEREST) Investigators. Effects of oral tolvaptan in patients hospitalized for worsening heart failure: The EVEREST Outcome Trial. JAMA J. Am. Med. Assoc..

[91] McMurray J.J., Teerlink J.R., Cotter G., Bourge R.C., Cleland J.G., Jondeau G., Krum H., Metra M., O’Connor C.M., Parker J.D. (2007). VERITAS Investigators. Effects of tezosentan on symptoms and clinical outcomes in patients with acute heart failure: The VERITAS randomized controlled trials. JAMA J. Am. Med. Assoc..

[92] Kjekshus J., Apetrei E., Barrios V., Böhm M., Cleland J.G., Cornel J.H., Dunselman P., Fonseca C., Goudev A., Grande P. (2007). Rosuvastatin in older patients with systolic heart failure. N. Engl. J. Med..

[93] Torre-Amione G., Anker S.D., Bourge R.C., Colucci W.S., Greenberg B.H., Hildebrandt P., Keren A., Motro M., Moyé L.A., Otterstad J.E. (2008). Results of a non-specific immunomodulation therapy in chronic heart failure (ACCLAIM trial): A placebo-controlled randomised trial. Lancet.

[94] Redfield M.M., Borlaug B.A., Lewis G.D., Mohammed S.F., Semigran M.J., Lewinter M.M., Deswal A., Hernandez A.F., Lee K.L., Braunwald E. (2012). PhosphdiesteRasE-5 Inhibition to Improve CLinical Status and EXercise Capacity in Diastolic Heart Failure (RELAX) trial: Rationale and design. Circ. Heart Fail..

[95] Gheorghiade M., Böhm M., Greene S.J., Fonarow G.C., Lewis E.F., Zannad F., Solomon S.D., Baschiera F., Botha J., Hua T.A. (2013). Effect of aliskiren on postdischarge mortality and heart failure readmissions among patients hospitalized for heart failure: The ASTRONAUT randomized trial. JAMA J. Am. Med. Assoc..

[96] Study to Evaulate the Safety and Efficacy of IV Infusion Treatment with Omecamtiv Mecarbil in Subjects with Left Ventricular Systolic Dysfunction Hospitalised for Acute Heart Failure (ATOMIC-AHF). www.Clinicaltrials.gov.

[97] Felker G.M., Teerlink J.R., Butler J., Hernandez A.F., Miller A.B., Cotter G., Davison B.A., Filippatos G., Greenberg B.H., Ponikowski P. (2014). Effect of serelaxin on mode of death in acute heart failure: Results from the RELAX-AHF study. J. Am. Coll. Cardiol..

[98] McMurray J.J., Packer M., Desai A.S., Gong J., Lefkowitz M.P., Rizkala A.R., Rouleau J.L., Shi V.C., Solomon S.D., Swedberg K. (2014). Angiotensin-neprilysin inhibition *versus* enalapril in heart failure. N. Engl. J. Med..

[99] Pieske B., Butler J., Filippatos G., Lam C., Maggioni A.P., Ponikowski P., Shah S., Solomon S., Kraigher-Krainer E., Samano E.T. (2014). Rationale and design of the SOluble guanylate Cyclase stimulatoR in heArT failurE Studies (SOCRATES). Eur. J. Heart Fail..

[100] Bardy G.H., Lee K.L., Mark D.B., Poole J.E., Packer D.L., Boineau R., Domanski M., Troutman C., Anderson J., Johnson G. (2005). Amiodarone or an implantable cardioverter-defibrillator for congestive heart failure. N. Engl. J. Med..

[101] Whellan D.J., Ousdigian K.T., Al-Khatib S.M., Pu W., Sarkar S., Porter C.B., Pavri B.B., O’Connor C.M., PARTNERS Study Investigators (2010). Combined heart failure device diagnostics identify patients at higher risk of subsequent heart failure hospitalizations: Results from PARTNERS HF (Program to Access and Review Trending Information and Evaluate Correlation to Symptoms in Patients with Heart Failure) study. J. Am. Coll. Cardiol..

[102] Assmus B., Fischer-Rasokat U., Honold J., Seeger F.H., Fichtlscherer S., Tonn T., Seifried E., Schächinger V., Dimmeler S., Zeiher A.M. (2007). Transcoronary transplantation of functionally competent BMCs is associated with a decrease in natriuretic peptide serum levels and improved survival of patients with chronic postinfarction heart failure: Results of the TOPCARE-CHD Registry. Circ. Res..

[103] Bolli R., Chugh A.R., D’Amario D., Loughran J.H., Stoddard M.F., Ikram S., Beache G.M., Wagner S.G., Leri A., Hosoda T. (2011). Cardiac stem cells in patients with ischaemic cardiomyopathy (SCIPIO): Initial results of a randomised phase 1 trial. Lancet.

[104] Trachtenberg B., Velazquez D.L., Williams A.R., McNiece I., Fishman J., Nguyen K., Rouy D., Altman P., Schwarz R., Mendizabal A. (2011). Rationale and design of the Transendocardial Injection of Autologous Human Cells (bone marrow or mesenchymal) in Chronic Ischemic Left Ventricular Dysfunction and Heart Failure Secondary to Myocardial Infarction (TAC-HFT) trial: A randomized, double-blind, placebo-controlled study of safety and efficacy. Am. Heart J..

[105] Perin E.C., Willerson J.T., Pepine C.J., Henry T.D., Ellis S.G., Zhao D.X., Silva G.V., Lai D., Thomas J.D., Kronenberg M.W. (2012). Effect of transendocardial delivery of autologous bone marrow mononuclear cells on functional capacity, left ventricular function, and perfusion in chronic heart failure: The FOCUS-CCTRN trial. JAMA J. Am. Med. Assoc..

[106] Hare J.M., Fishman J.E., Gerstenblith G., DiFede Velazquez D.L., Zambrano J.P., Suncion V.Y., Tracy M., Ghersin E., Johnston P.V., Brinker J.A. (2012). Comparison of allogeneic vs autologous bone marrow–derived mesenchymal stem cells delivered by transendocardial injection in patients with ischemic cardiomyopathy: The POSEIDON randomized trial. JAMA J. Am. Med. Assoc..

[107] Makkar R.R., Smith R.R., Cheng K., Malliaras K., Thomson L.E., Berman D., Czer L.S., Marbán L., Mendizabal A., Johnston P.V. (2012). Intracoronary cardiosphere-derived cells for heart regeneration after myocardial infarction (CADUCEUS): A prospective, randomised phase 1 trial. Lancet.

[108] Vrtovec B., Poglajen G., Lezaic L., Sever M., Socan A., Domanovic D., Cernelc P., Torre-Amione G., Haddad F., Wu J.C. (2013). Comparison of transendocardial and intracoronary CD34+ cell transplantation in patients with nonischemic dilated cardiomyopathy. Circulation.

[109] Karantalis V., DiFede D.L., Gerstenblith G., Pham S., Symes J., Zambrano J.P., Fishman J., Pattany P., McNiece I., Conte J. (2014). Autologous mesenchymal stem cells produce concordant improvements in regional function, tissue perfusion, and fibrotic burden when administered to patients undergoing coronary artery bypass grafting: The Prospective Randomized Study of Mesenchymal Stem Cell Therapy in Patients Undergoing Cardiac Surgery (PROMETHEUS) trial. Circ. Res..

[110] Bartunek J., Davison B., Sherman W., Povsic T., Henry T.D., Gersh B., Metra M., Filippatos G., Hajjar R., Behfar A. (2016). Congestive Heart Failure Cardiopoietic Regenerative Therapy (CHART-1) trial design. Eur. J. Heart Fail..

[111] Alrefai M.T., Murali D., Paul A., Ridwan K.M., Connell J.M., Shum-Tim D. (2015). Cardiac tissue engineering and regeneration using cell-based therapy. Stem Cells Cloning.

[112] Blau H.M., Brazelton T.R., Weimann J.M. (2001). The evolving concept of a stem cell: Entity or function?. Cell.

[113] Dib N., Dinsmore J., Lababidi Z., White B., Moravec S., Campbell A., Rosenbaum A., Seyedmadani K., Jaber W.A., Rizenhour C.S. (2009). One-year follow-up of feasibility and safety of the first U.S., randomized, controlled study using 3-dimensional guided catheter-based delivery of autologous skeletal myoblasts for ischemic cardiomyopathy (CAuSMIC study). JACC Cardiovasc. Interv..

[114] Patel A.N., Geffner L., Vina R.F., Saslavsky J., Urschel H.C., Kormos R., Benetti F. (2005). Surgical treatment for congestive heart failure with autologous adult stem cell transplantation: A prospective randomized study. J. Thorac. Cardiovasc. Surg..

[115] Heldman A.W., DiFede D.L., Fishman J.E., Zambrano J.P., Trachtenberg B.H., Karantalis V., Mushtaq M., Williams A.R., Suncion V.Y., McNiece I.K. (2014). Transendocardial mesenchymal stem cells and mononuclear bone marrow cells for ischemic cardiomyopathy: The TAC-HFT randomized trial. JAMA J. Am. Med. Assoc..

[116] Freyman T., Polin G., Osman H., Crary J., Lu M., Cheng L., Palasis M., Wilensky R.L. (2006). A quantitative, randomized study evaluating three methods of mesenchymal stem cell delivery following myocardial infarction. Eur. Heart J..

[117] Moss J.A. (2014). Gene therapy review. Radiol. Technol..

[118] Williams M.L., Koch W.J. (2004). Viral-based myocardial gene therapy approaches to alter cardiac function. Annu. Rev. Physiol..

[119] Chira S., Jackson C.S., Oprea I., Ozturk F., Pepper M.S., Diaconu I., Braicu C., Raduly L.Z., Calin G.A., Berindan-Neagoe I. (2015). Progresses towards safe and efficient gene therapy vectors. Oncotarget.

[120] Svensson E.C., Marshall D.J., Woodard K., Lin H., Jiang F., Chu L., Leiden J.M. (1999). Efficient and stable transduction of cardiomyocytes after intramyocardial injection or intracoronary perfusion with recombinant adeno-associated virus vectors. Circulation.

[121] Giacca M., Zacchigna S. (2012). VEGF gene therapy: Therapeutic angiogenesis in the clinic and beyond. Gene Ther..

[122] Beeri R., Chaput M., Guerrero J.L., Kawase Y., Yosefy C., Abedat S., Karakikes I., Morel C., Tisosky A., Sullivan S. (2010). Gene delivery of sarcoplasmic reticulum calcium ATPase inhibits ventricular remodeling in ischemic mitral regurgitation. Circ. Heart Fail..

[123] Cannavo A., Liccardo D., Koch W.J. (2013). Targeting cardiac β-adrenergic signaling via GRK2 inhibition for heart failure therapy. Front. Physiol..

[124] Raake P.W., Tscheschner H., Reinkober J., Ritterhoff J., Katu H.A., Koch W.J., Most P. (2011). Gene therapy targets in heart failure: The path to translation. Clin. Pharmacol. Ther..

[125] World Health Organization ICD-10 Second Edition Volume 2. http://www.who.int/classifications/icd/ICD-10_2nd_ed_volume2.pdf.

[126] Hale D. (2009). ACP Hospitalist: Billing and Coding. http://www.acphospitalist.org/archives/2009/01/coding.htm#sb1.

